# Effects of Lactation Lactoferrin Deficiency on Intestinal Microbiota in Different Mice Models

**DOI:** 10.3390/nu17132248

**Published:** 2025-07-07

**Authors:** Wenli Wang, Qin An, Yunxia Zou, Qingyong Meng, Yali Zhang

**Affiliations:** 1 College of Food Science and Nutritional Engineering, China Agricultural University, Beijing 100083, China; wenliwang@sjtu.edu.cn (W.W.);; 2 The International Peace Maternity and Child Health Hospital, Shanghai Jiao Tong University School of Medicine, Shanghai 201400, China; 3 College of Biological Sciences, China Agricultural University, Beijing 100083, China

**Keywords:** lactation, lactoferrin, obesity, IBD, depression, gut microbiota

## Abstract

Background/Objectives: The establishment of early gut microbiota is crucial for host health. Lactoferrin (LF), which is present in breast milk, positively impacts gut microbiota composition. However, the effect of lactation LF on the establishment and composition of early gut microbiota in different disease models in adulthood remains unclear. Methods: Lactation-LF-deficient mice were established using systemically LF–knocked-out maternal mice. This study assessed the maturity of the gut microbiota in LF feeding-deficient mice in relation to age and changes in the gut microbiota in adult high-fat diet (HFD)-induced obesity, dextran sodium sulfate (DSS)-induced acute colitis, and chronic unpredictable mild stress (CUMS)-induced depression models. Results: Compared to LF intake during lactation, LF deficiency during lactation increased the abundance of potentially pathogenic bacteria in the gut, resulting in abnormal microbial maturation. LF intake during lactation aggravated gut microbiota dysbiosis induced via HFD, DSS, and CUMS in adulthood and may change the function of *Enterorhabdus*, *GCA-900066575*, *Peptococcus*, *Tuzzerella*, *Akkermansia*, and *Desulfovibrio*. Comparing the different models revealed that bacteria that were jointly upregulated via HFD and DSS exhibited increased levels of inflammation and oxidation. LF deficiency during lactation may weaken the association between an HFD and inflammatory bowel disease (IBD). The changing trends in many gut microbes caused by DSS and HFD were opposite to those that changed with age. Conclusions: Lactoferrin deficiency increases the abundance of potential pathogens and disrupts microbial maturation. This lack of LF exacerbates dysbiosis in models of obesity, colitis, and depression. Regulating the gut microbiota according to the rules of microbial succession during the maturation process of gut microbiota may improve gut microbiota dysbiosis in patients with obesity and IBD.

## 1. Introduction

In the gastrointestinal tract, the concentration of microbial cells reaches 10^12^ cells/mL, with a cumulative mass of one kilogram, serving as an additional bioreactor fueled by dietary macronutrients. Their genetic material significantly affects the physical development, metabolic processes, and overall well-being of the host and is intricately intertwined with their health and susceptibility to diseases [1]. Accumulating research has Zhao, Q. demonstrated that the establishment of gut microbiota in early life lays the foundation for the adult gut microbial community and plays a dominant role in shaping the host’s future health [2]. In both human and animal models, gut microbiome dysbiosis is closely linked to the development and worsening of obesity, inflammatory bowel disease (IBD), and major depressive disorders (MDDs) [2,3]. Epidemiological studies have shown that the excessive consumption of a high-fat diet (HFD) is strongly associated with IBD progression and the recurrence of IBD [4]. Furthermore, comorbid HFD and IBD with depressive syndromes have been reported in preclinical and clinical studies [5,6,7]. However, the role of gut microbes in the association between these diseases remains unclear.

Lactoferrin (LF), a vital bioactive protein, is abundantly found in human breast milk. Currently, formula serves as the primary alternative to breast milk, yet formula is not a complete substitute for breast milk because LF is not widely added. Therefore, infants fed formula milk may be at risk of LF deficiency or insufficiency during the lactation period. LF plays an active role in the establishment of intestinal microorganisms in early life [8], Wang et al. predicted that LF may have the potential to prevent the occurrence of chronic diseases such as obesity, IBD, and depression in adulthood by influencing the establishment of early microorganisms [8]. However, the effects of lactating LF on the development of intestinal microbes and the composition of intestinal microbes in different adult disease models remain unclear. This study determined the effects of lactational LF deficiency on the development of intestinal microbial composition in mice and the changes in HFD, dextran sodium sulfate (DSS)-induced acute colitis, and chronic unpredictable mild stress (CUMS)-induced depression models and summarized the relationship between these models of intestinal microbes. This study aimed to provide a novel therapeutic strategy and a theoretical basis for intestinal flora intervention in patients with obesity and IBD.

## 2. Method

### 2.1. Animal

**Figure 1:**
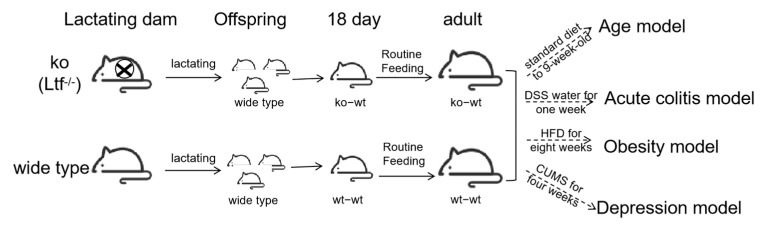
Study design schematic.

Heterozygous *Ltf* knockout (KO) mice were generated by Biocytogen Co., Ltd. (Beijing, China). Through heterozygous breeding, *Ltf* KO and wild-type (WT) littermates on a C57BL/6N background were produced, followed by homozygous breeding to obtain sufficient age-matched WT and KO mice for experiments. Prior to the study, synchronized breeding of KO and WT mice was performed. Within 48 h postpartum, all pups from WT dams were cross-fostered to KO dams (ko-wt group), while control litters remained with WT dams (wt-wt group). At weaning (day 21), offspring were sex-segregated into cages (5 mice/cage). The ko-wt group received LF-deficient milk during lactation, and both groups (wt-wt and ko-wt) were subsequently used for experimental analyses. The study design schematic is as follows (Figure 1).

Mice were maintained under standardized conditions (standard 12 h:12 h light–dark cycle, 22 °C ± 2 °C, humidity: 55 ± 10%) with free access to food and water. At the end of the experiment, mice were anesthetized via an intraperitoneal injection of 1% pentobarbital sodium (40 mg/kg) and subjected to blood collection via orbital puncture. Euthanasia was then performed via cervical dislocation. Minimum sample sizes for quantitative data were determined using the resource equation method and following the 3R principle (Replacement, Reduction, Refinement). The minimum sample size was calculated as follows: min *n* = E/k + 1 (minimum E = 10), where E represents the degrees of freedom for an ANOVA, k denotes the number of groups, and *n* indicates the sample size per group. Age model: k = 3 → min *n* = 5; obesity model and acute colitis model: k = 4 → min *n* = 4; Depression model: k = 2 → min *n* = 6.

As this is a fundamental research study without regulatory purposes, registration was not required per international guidelines (e.g., ICLAS recommendations). Our study design complied with the ARRIVE 2.0 guidelines and China’s Regulations for the Administration of Affairs Concerning Experimental Animals, and it followed ethical standards approved by the Institutional Animal Care and Use Committee (SYXK 2020-0052), with official authorization from the Animal Experimentation Committee of China Agricultural University (Approval Code: AW40702202-4-6; Approval Date: 4 July 2022).

#### 2.1.1. Age Model

Eighteen-day-old mice (ko-wt-18day, *n* = 8; wt-wt-18day, *n* = 8), 9-week-old female mice (wt-wt-9week-female, *n* = 8; ko-wt-9week-female, *n* = 8), 9-week-old male mice (wt-wt-9week-male, *n* = 7; ko-wt-9week-male, *n* = 8) were housed with free access to standard feed and water (a total of 47 mice), and their cecal samples were harvested and rapidly frozen using liquid nitrogen.

#### 2.1.2. High-Fat Diet Model

In this study, a total of 28 seven-week-old ko-wt male mice and wt-wt male mice with comparable body weights were randomly selected. These two groups were further divided into four groups: control diet-fed wt-wt group mice (Conwt-wt group) (*n* = 4), control diet-fed ko-wt group mice (Conko-wt group) (*n* = 4), high-fat diet-fed wt-wt group (HFDwt-wt group) (*n* = 10), and high-fat diet-fed ko-wt group mice (HFDko-wt group) (*n* = 10). The body weight was measured weekly. After 8 weeks of dietary intervention, the mice were fasted for 8 h and then euthanized to obtain blood samples by enucleating the eyeballs. The blood samples were then separated into serum samples via centrifugation at 5000 rpm for 15 min at 4 °C. Cecal samples were harvested and rapidly frozen using liquid nitrogen.

The control diet (Con) provided 10% of energy from fat, 20% from protein, and 70% from carbohydrates, with a calorific value of 3.42 kcal/g. The HFD provided 60% of energy from fat, 20% from protein, and 20% from carbohydrates, with a calorific value of 5.24 kcal/g, and this feed was purchased from Jiangsu Synergy Pharmaceutical Biotechnology Co., Ltd. (Nanjing, China).

#### 2.1.3. DSS-Induced Colitis

Eight-week-old male (mDWT-WT, *n* = 7; mDKO-WT, *n* = 8) and female mice (fDWT-WT, *n* = 8; fDKO-WT, *n* = 6) were subjected to an oral administration of 2.5% *w*/*v* DSS (MW = 36,000–50,000 kDa; MP Biomedicals, LLC, Irvine, CA, USA) in their drinking regimen for a duration of 7 days. As a control group, 8-week-old male (mWT-WT, *n* = 7; mKO-WT, *n* = 8) and female mice (fWT-WT, *n* = 8; fKO-WT, *n* = 8) were allowed access to regular drinking water for the same period (a total of 60 mice). The control group here corresponds to the same cohort of mice as the wt-wt-9week-male and ko-wt-9week-male groups in the age model. Cecal samples were harvested and rapidly frozen using liquid nitrogen [9].

#### 2.1.4. CUMS-Induced Depression-like Behavior

Nine-week-old male mice (CUMSwt-wt, *n* = 8; CUMSko-wt, *n* = 8) were selected for a four-week CUMS depression model (a total of 16 mice). The CUMS model was applied as previously proposed by Wang [9]. The CUMS detail and schedule are presented in Table S1.

### 2.2. Microbiome Composition Assessment via 16S rRNA Amplicon Sequencing

The experimental procedures were performed according to methods established by Wang [9].

### 2.3. Statistical Analysis

The data are presented as means ± standard errors of the means. Student’s *t*-test was used to compare two groups. A one-way ANOVA analysis and Tukey’s analysis were used for the statistical significance of the differences among groups. Analyses of the 16S rRNA microbiome sequencing data were performed using the online platform of Majorbio Cloud Platform. All data were obtained using Majorbio Cloud Platform (cloud.majorbio.com) and GraphPad Prism 10.2.0 software (San Diego, CA, USA). A *p*-value < 0.05 was considered statistically significant (* *p* < 0.05, ** *p* < 0.01, and *** *p* < 0.001); different letters stand for statistically significantly different from each other. All analyses of animal studies’ results were conducted blindly.

## 3. Results

### 3.1. Alterations in the Composition of the Intestinal Microbiome with Age in Mice

**Figure 2:**
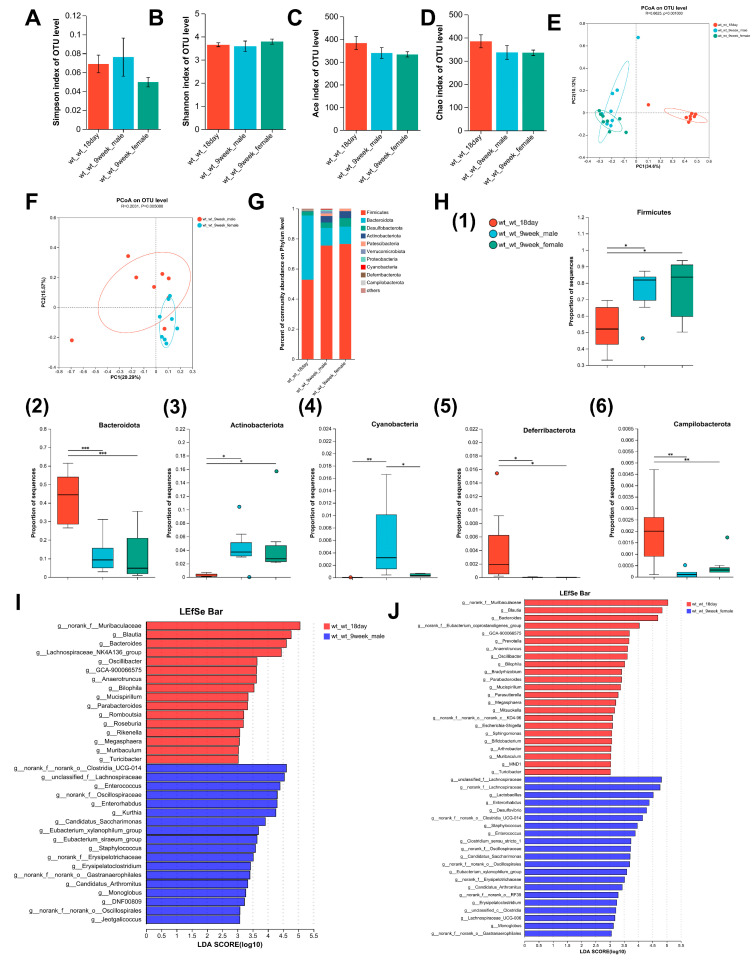
Changes in intestinal microbiome composition with age in mice. (**A**–**D**) Comparison of the Simpson index, the Shannon index, the Ace index, and the Chao index of intestinal microbiota between 9-week-old mice and 18-day-old mice. (**E**) PCoA of bacterial β-diversity based on the Bray–Curtis dissimilarity index in the age model and evaluated using ANOSIM. (**F**) PCoA of bacterial β-diversity based on the Bray–Curtis dissimilarity index in adult male and female mice and evaluated using ANOSIM. (**G**) Percentage of community abundance at the phylum level in 18-day-old mice and 9-week-old mice. (**H**) Comparison of the proportion of bacterial phylum (1–6) Firmicutes, Bacteroidota, Actinobacteriota, Cyanobacteria, Deferribacterota, and Campilobacterota in the gut microbiota between 18-day-old and 9-week-old mice. (**I**) Linear discriminant analysis (LDA) scores derived from LEfSe-analysis at the genus level in the wt-wt-18 day group and the wt-wt-9week-male group. (**J**) LDA scores derived from LEfSe-analysis at the genus level in the wt-wt-18day group and the wt-wt-9week-female group. LDA > 3, *n* = 8–9. (**A**–**D**,**H**) was evaluated using a one-way ANOVA, post hoc with Tukey’s test, * *p* < 0.05, ** *p* < 0.01, *** *p* < 0.001. *n* = 8–9.

α diversity represents the richness and diversity of the microbial communities. The Simpson and Shannon indices are measures of community diversity, with the Simpson index reflecting negative correlations and the Shannon index reflecting positive correlations with community diversity. The Chao and ACE indices were positively correlated with community richness. As illustrated in Figure 2A–D, there was no significant difference in parameters characterizing the richness and diversity of gut microbes in relation to age, suggesting that the α diversity of the mice gut microbes did not change significantly with an increasing age. The β diversity was markedly different in the gut microbiome composition between 18-day-old and adult mice (Figure 2E), and a significant difference was observed in the gut microbiome composition between female and male adult mice (Figure 2F). Figure 2G illustrates the distribution of intestinal microbes at the phylum level. In 18-day-old mice, the combined abundance of Firmicutes, Bacteroidetes, Desulfobacteria, and Actinobacteria accounted for 98.36% of the total, whereas in adult male and female mice, the combined abundance of the aforementioned phyla was 95.05% and 98.38%, respectively.

Adult mice displayed a notable increase in Firmicutes and Actinobacteria populations relative to 18-day-old mice. Conversely, the abundance of Bacteroidetes, Deferribacterota, and Campilobacterota was significantly lower in adult mice than in 18-day-old mice, and a significant elevation in Cyanobacteria levels was observed in adult male mice relative to both 18-day-old pups and the adult female group (Figure 2H). Changes in intestinal microorganisms were further evaluated at the genus level with respect to age. LEfSe analysis identified 34 taxonomic biomarkers in wt_wt_18day mice and wt_wt_9week_male mice with an LDA score > 3 and *p* < 0.05, respectively (Figure 2I). With increasing age, the abundances of 16 genera decreased, whereas those of 18 genera increased in wt-wt adult male mice. In adult female mice, LEfSe analysis identified 44 taxa biomarkers in wt_wt_18day mice and wt_wt_9week_female mice (Figure 2J). Twenty-three genera showed a significant increase compared to 18-day-old mice. Conversely, the abundance of 21 genera exhibited a significant decrease compared to 18-day-old mice. With increasing age, there was a decrease in the abundance of 11 bacterial taxa, namely, *Blautia*, *Bacteroides Anaerotruncus*, *Bilophila*, *GCA-900066575*, *Megasphaera*, *Mucispirillum*, *Muribaculum*, *Oscillibacter*, *Parabacteroides*, and *Turicibacter* in both male and female mice. The proportion of eight bacterial categories showed an increase, including *Candidatus_Arthromitus*, *Candidatus_Saccharimonas*, *Enterococcus*, *Enterorhabdus*, *Erysipelatoclostridium*, *Eubacterium_xylanophilum_group*, *Monoglobus*, and *Staphylococcus* in both sexes of mice.

### 3.2. Alterations in the Composition of the Intestinal Microbiome with Age in Lactation LF Feeding-Deficient Mice

**Figure 3:**
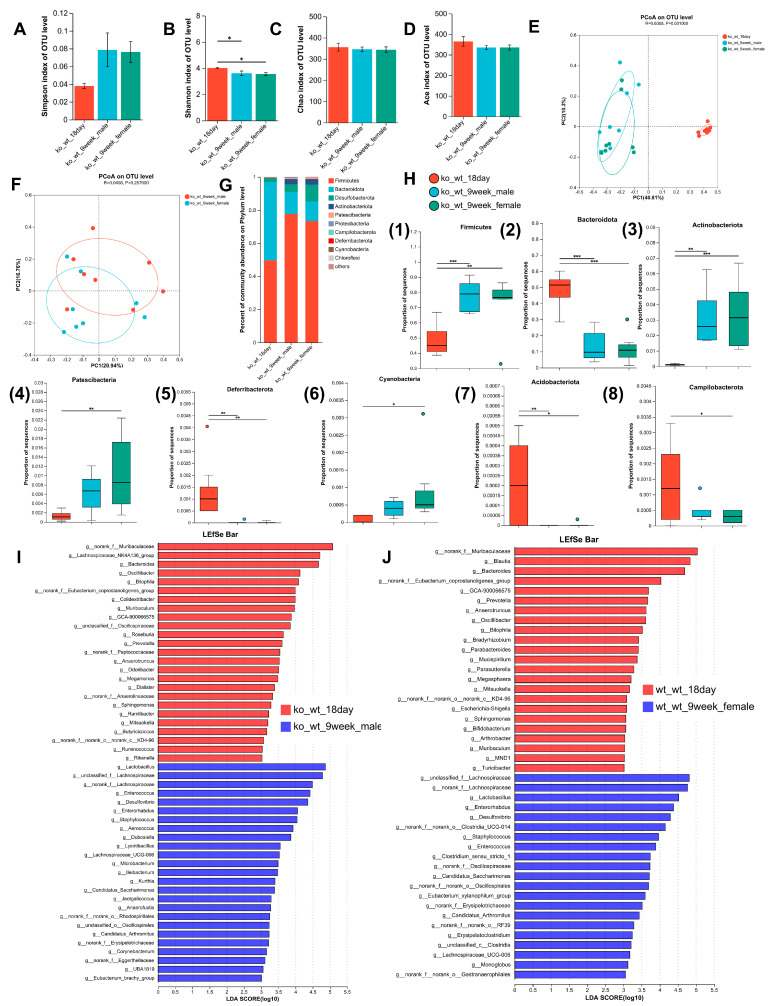
Changes in the intestinal microbiome composition with age in lactation LF-deficient mice. (**A**–**D**) Comparison of the Simpson index, the Shannon index, the Ace index, and the Chao index of intestinal microbiota between 9-week-old lactation LF-deficient mice and 18-day-old lactation LF-deficient mice. (**E**) PCoA of bacterial β-diversity based on the Bray–Curtis dissimilarity index in lactation LF-deficient age model and evaluated using ANOSIM. (**F**) PCoA of bacterial β-diversitybased on the Bray–Curtis dissimilarity index in adult male and female lactation LF-deficient mice and evaluated using ANOSIM. (**G**) Percentage of community abundance on the phylum level in 18-day-old and 9-week-old lactation LF-deficient mice. (**H**) Comparison of the proportion of bacterial phylum (1–8) Firmicutes, Bacteroidota, Actinobacteriota, Patescibacteria, Deferribacterota, Cyanobacteria, Acidobacteriota and Campilobacterota in the gut microbiota between 18-day-old and 9-week-old lactation LF-deficient mice. (**I**) LDA scores derived from LEfSe analysis at the genus level in ko-wt-18day group and ko-wt-9week-male group. (**J**) LDA scores derived from LEfSe analysis at the genus level in ko-wt-18day group and ko-wt-9week-female group. LDA > 3. (**A**–**E**,**H**) were evaluated using a one-way ANOVA, post hoc with Tukey’s test, * *p* < 0.05, ** *p* < 0.01, *** *p* < 0.001. *n* = 8–9.

The Shannon index was markedly lower in adult mice relative to 18-day-old mice, although no significant variations were observed in the Simpson, Chao, or Ace indices (Figure 3A–D). These results indicated that the intestinal microbial community diversity of lactating LF-deficient adult mice decreased significantly compared to that of 18-day-old mice, while richness showed no significant variation. The β diversity of the gut microbiome exhibited significant differences between 18-day-old and adult models (Figure 3E), but no significant difference was observed between female and male adult mice (Figure 3F). Figure 3G shows the distribution of intestinal microbes at the phylum level in 18-day-old adult male and female lactating, LF-deficient mice. In 18-day-old mice, the total abundance of Firmicutes, Bacteroidetes, and Desulfobacterota was 99.26%. The total abundance of Firmicutes, Bacteroidetes, and Desulfobacterota in adult male and female mice was 95.55% and 95.42%, respectively.

In Figure 3H, there was a significant increase in Firmicutes and Actinobacteria in adult lactating LF-deficient mice compared to their 18-day-old counterparts. Conversely, the abundance of Bacteroidetes, Deferribacterota, and Acidobacteria was significantly lower in adult lactating LF-deficient mice than in 18-day-old lactating LF-deficient pups. Notably, in adult female lactating LF-deficient mice, there was a notable increase in the abundance of Patescinacteria and a significant decrease in Cyanobacteria and Campylobacterota abundance compared to 18-day-old lactating LF-deficient mice. The LEfSe Bar in Figure 3I shows the predominant genus in LF-deficient 18-day-old mice and adult male mice. With increasing age, the abundance of 25 genera decreased, whereas 25 genera increased in male mice. As shown in Figure 3J, the abundances of 25 genera decreased, whereas those of 19 genera increased in female mice. In the absence of LF during lactation, the abundance of 15 bacterial taxa including *Anaerotruncus*, *Bacteroides*, *Bilophila*, *Butyricicoccus*, *Colidextribacter*, *Dialister*, *GCA-900066575*, *Megamonas*, *Muribaculum*, *Odoribacter*, *Oscillibacter*, *Prevotella*, *Ramlibacter*, *Ruminococcus*, and *Sphingomonas* gradually decreases with age in both male and female mice. Conversely, the abundance of eight taxa including *Candidatus_Arthromitus*, *Candidatus_Saccharimonas*, *Desulfovibrio*, *Dubosiella*, *Enterococcus*, *Enterorhabdus*, *Eubacterium_brachy_group*, and *Lactobacillus* gradually increased with age, regardless of the sex of the mice.

To achieve a deeper insight into the functional diversity exhibited by the microbial populations within the samples, we conducted functional predictions of the bacterial data using BugBase. In LF-intake-normal mice during lactation, we observed a significant decrease in the ‘gram-negative’ phenotype and a significant increase in the ‘Contains-Mobile-Elements’, ‘Forms-Biofilms’, and ‘Facultatively Anaerobic’ phenotypes in the gut microbiota of adult mice (both sexes) compared to 18-day-old mice. The ‘gram-positive’ phenotype only showed an increase in male mice, whereas the ‘aerobic’ phenotype only increased in female mice (Figure S1A,B). In LF-deficient mice during lactation, we observed a significant decrease in the ‘potentially pathogenic’ and ‘gram-negative’ phenotypes and a significant increase in the ‘forms-biofilms’ and ‘facultatively anaerobic’ phenotypes when comparing adult mice (both sexes) to 18-day-old mice; the ‘gram-positive’ phenotype only increased in female mice, whereas the ‘aerobic’ phenotype only increased in males (Figure S1C,D).

### 3.3. Effects of LF Deficiency During Lactation on Intestinal Microbial Composition in 18-Day-Old and Adult Mice

**Figure 4:**
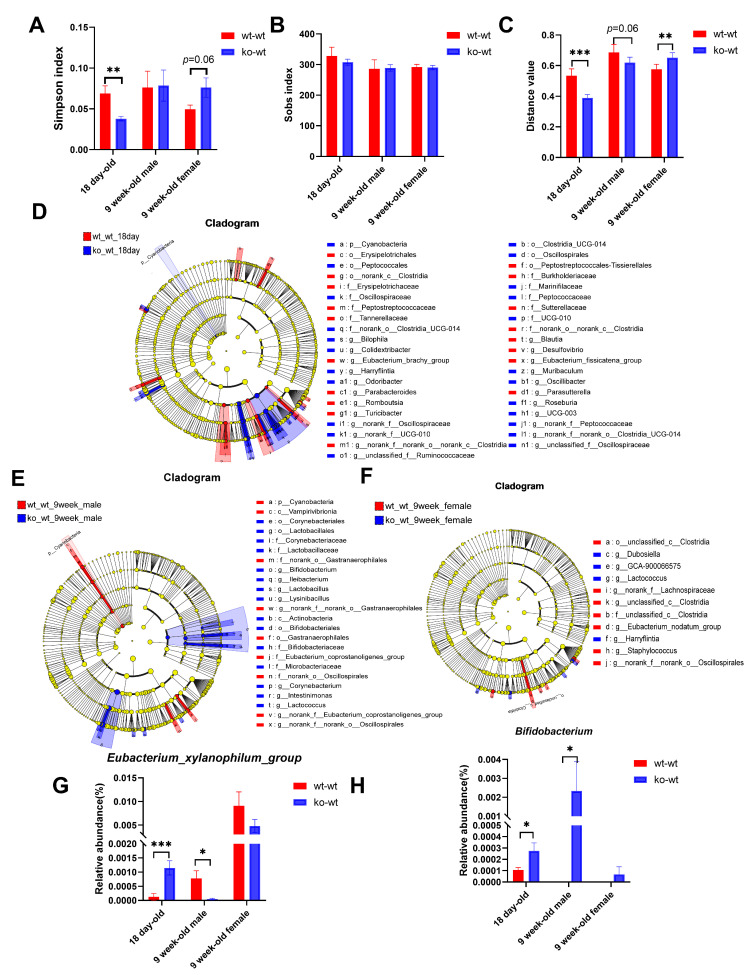
Effects of LF during lactation on intestinal microbial composition of 18-day-old and adult healthy mice. (**A**) Simpson index. (**B**) Sob index difference analysis between LF-intake and LF-deficient mice in 18-day-old and adult healthy mice. (**C**) β diversity difference analysis between LF-intake and LF-deficient mice in 18-day-old and adult healthy mice. (**D**) LEfSe comparison between wt_wt_18 day and ko_wt_18day mice from the phylum to genus level. (**E**) LEfSe comparison between wt_wt_9week and ko_wt_9week male mice from the phylum level to the genus level. (**F**) LEfSe comparison between wt_wt_9week and ko_wt_9week female mice from the phylum to genus level. LDA > 3. (**G**) Comparison of the relative abundance of the *Eubacterium_xylanophilum_group* and (**H**) *Bifidobacterium* between LF-intake and LF-deficient mice in 18-day-old and adult healthy mice. (**A**–**C**,**G**,**H**) were evaluated using Student’s-*t*-test, * *p* < 0.05, ** *p* < 0.01, *** *p* < 0.001. *n* = 8–9.

As shown in Figure 4A,B, LF deficiency during lactation significantly reduced the Simpson index in 18-day-old mice and increased the Simpson index in adult female mice, with no effect on the Sob index in 18-day-old and adult mice. This indicates that the LF deficiency during lactation leads to an increase in the community diversity of the gut microbiota in 18-day-old mice but a decrease in adult female mice and has no effect on the richness in both 18-day-old and adult mice. β diversity analysis revealed that lactating LF deficiency led to substantial alterations in gut microbiota composition compared to normal LF consumption in both 18-day-old and 9-week-old mice (Figure 4C). As shown in Figure 4D, lactating LF deficiency significantly increased the Cyanobacteria level in 18-day-old mice and a total of 23 bacterial genera, with significant differences between wt_wt_18day and ko_wt_18day groups. As shown in Figure 4E, LF deficiency during lactation significantly decreased the abundance of the Cyanobacteria in 9-week-old mice, and a total of 7 families and 10 genera with significant differences were screened between the wt_wt_9week male and ko_wt_9week male groups. As shown in Figure 4F, nine genera with significant differences were screened between the wt_wt_9week female and ko_wt_9week female groups.

Comparing the gut microbial phenotypes of ko-wt18day mice with those of wt-wt18day mice, we observed an increase in potentially pathogenic microbial populations due to LF deficiency during lactation (Figure S1E), which persisted in adult female mice but disappeared in male mice (Figure S1F,G).

### 3.4. Effects of LF Deficiency During Lactation on Intestinal Microbial Composition in Adult Obese Mice Induced by HFD

After stimulation with a high-fat diet for 8 weeks, the body weight of HFDwt-wt mice significantly increased and was 28% higher than that of the Conwt-wt group, indicating the successful modeling of obesity (unpublished results). The measurement of serum lipoprotein levels showed that HFD significantly increased serum total cholesterol (TC), high-density lipoprotein (HDL), and low-density lipoprotein (LDL) levels but had no significant effect on serum triglyceride (TG) levels (unpublished results). Moreover, the HFDko-wt group showed significantly higher serum LDL and vLDL levels than the HFDwt-wt group (unpublished results). These results indicated that LF-deficient mice exhibited worse serum lipoprotein levels following HFD stimulation during lactation.

**Figure 5:**
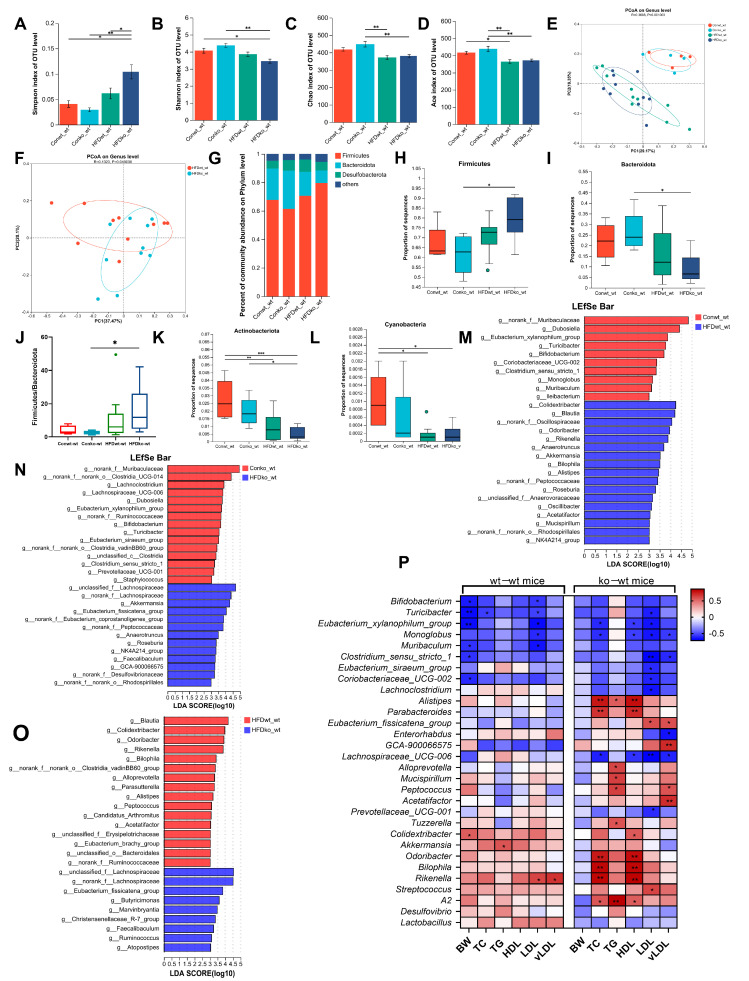
The influence of HFD on the composition of the gut microbiota in lactation LF-feeding mice and LF-deficient mice. (**A**–**D**) The impact of HFD on the gut microbiota Simpson index, the Shannon index, the Ace index, and the Chao index in lactation LF-feeding mice and LF-deficient mice. (**E**) PCoA of bacterial β-diversity based on the Bray–Curtis dissimilarity index in the HFD model and evaluated using ANOSIM. (**F**) PCoA of bacterial β-diversity based on the Bray–Curtis dissimilarity index in HFDko-wt and HFDwt-wt groups and evaluated using ANOSIM. (**G**) Percentage of community abundance on phylum level in the Conwt-wt, Conko-wt, HFDwt-wt, and HFDko-wt groups. (**H**,**I**) The impact of HFD on the abundance of Firmicutes, Bacteroides in lactation LF-feeding mice and LF-deficient mice. (**J**) Firmicutes/Bacteroidetes ratio in the Conwt-wt, Conko-wt, HFDwt-wt, and HFDko-wt groups. (**K**,**L**) The impact of HFD on the abundance of Actinobacteriota and Cyanobacteria in lactation LF-feeding mice and LF-deficient mice. (**M**) LDA scores derived from LEfSe analysis at the genus level in the Conwt-wt group and the HFDwt-wt group. (**N**) LDA scores derived from LEfSe analysis at the genus level in the Conko-wt group and the HFDko-wt group. (**O**) LDA scores derived from LEfSe analysis at the genus level in the HFDwt-wt group and the HFDko-wt group. (**P**) Pearson correlation analysis between genera of gut microbiota and obesity phenotype in LF-feeding and LF-deficient mice. LDA > 3, (**A**–**D**,**H**–**L**) were evaluated using two-way ANOVA, * *p* < 0.05, ** *p* < 0.01, *** *p* < 0.001. *n* = 4–10.

As shown in Figure 5A,B, in lactating LF-deficient mice, HFD significantly increased the Simpson index and decreased the Shannon index compared to normal diet-fed mice. However, in mice with standard LF intake, HFD feeding did not alter microbial diversity (Simpson/Shannon indices) compared to controls. Additionally, among HFD-fed obese mice, the HFDko-wt group exhibited a significantly elevated Simpson index relative to the HFDwt-wt group. These results suggest that LF deficiency during lactation aggravates the decrease in intestinal microbial diversity induced via HFD. The Chao index of the HFDko-wt group was significantly lower than that of the Conko-wt group, as shown in Figure 5C. However, there was no significant difference in the Chao index between the HFDwt-wt and Conwt-wt groups. The results in Figure 5D suggest that HFD significantly reduced the Ace index in lactating LF-deficient mice and lactating LF normal intake mice compared to a normal diet. This indicated that an HFD significantly reduced the richness of the intestinal microbial composition in adult mice, regardless of LF intake. As shown in Figure 5E, the composition of the intestinal microflora in the HFDwt-wt and HFDko-wt groups was significantly different from that in the Conwt-wt and Conko-wt groups, indicating that the composition of the intestinal microflora in adult mice changed significantly after HFD treatment. To further explore the effect of LF-deprivation during early life on intestinal microorganisms in adult obese mice induced via HFD, we analyzed the β diversity of intestinal microorganisms in HFDwt-wt and HFDko-wt groups. As shown in Figure 5F, the intestinal microorganisms in the HFDwt-wt and HFDko-wt groups were significantly separated at the genus level, indicating that the loss of LF during lactation affected the composition of intestinal microorganisms in adult mice stimulated via HFD.

To explore the species composition of the microbial communities in the different groups, a histogram of the microbial community was drawn according to the results of the taxonomic analysis. A bar map of the intestinal microbial community was drawn at the phylum level, and the results showed that Firmicutes, Bacteroides, and Desulfobacterota were the dominant phyla in all mice (Figure 5G). As shown in Figure 5H,I, the abundance of Firmicutes and Bacteroidetes in the HFDwt-wt group was not different from that in the Conwt-wt group; however, the abundance of Firmicutes increased, and that of Bacteroidetes decreased significantly in the HFDko-wt group compared to the Conko-wt group. A further analysis of the Firmicutes to Bacteroidetes (F/B) ratio is shown in Figure 5J. Compared with the Conwt-wt group, F/B in HFDwt-wt group was increased, but there was no significant difference, and the F/B ratio in the HFDko-wt group was significantly higher than that in the Conko-wt group. Figure 5K shows that HFD increased the abundance of Actinobacteria, regardless of the intake of LF during lactation. The abundance of Cyanobacteria in HFDwt-wt group was significantly higher than that in the Conwt-wt group (Figure 5L). This suggests that mice without LF intake during lactation are more likely to show changes in the composition of the intestinal flora under HFD stimulation.

Figure 5M shows the effect of HFD on intestinal microflora in mice with normal LF intake. We found that the abundance of *Dubosiella*, *Turicibacter*, *Bifidobacterium*, *Eubacterium xylanophilum group*, *Coriobacteriaceae UCG-002*, *Clostridium sensu stricto 1*, *Monoglobus*, *Muribaculum*, and *Ileibacterium* decreased significantly, which showed a negative correlation with serum LDL levels (Figure 5P), and *Colidextribacter*, *Blautia*, *Odoribacter*, *Rikenella*, *Anaerotruncus*, *Alistipes*, *Bilophila*, *Akkermansia*, *Roseburia*, *NK4A214_group*, *Oscillibacter*, *Mucispirillum*, and *Acetatifactor* increased significantly in the HFDwt-wt group. In lactating LF-deficient mice (Figure 5N), HFD significantly decreased the abundance of *Lachnoclostridium*, *Lachnospiraceae_UCG-006*, *Dubosiella*, *Eubacterium_xylanophilum_group*, *Bifidobacterium*, *Turicibacter*, *Eubacterium_siraeum_group*, *Clostridium_sensu_stricto_1*, *Prevotellaceae_UCG-001*, and *Staphylococcus*, and it increased the abundance of *Roseburia*, *Akkermansia*, *Eubacterium_fissicatena_group*, *Faecalibaculum*, *Anaerotruncus*, *NK4A214_group*, and *GCA-900066575*. The relative abundances of five genera, *Bifidobacterium*, *Clostridium_sensu_stricto_1*, *Dubosiella*, *Eubacterium_xylanophilum_group*, and *Turicibacter*, decreased, and the relative abundances of four genera, *Akkermansia*, *Anaerotruncus*, *NK4A214_group*, and *Roseburia*, increased in both LF-feeding and LF-lacking mice. In Figure 5O, the abundance of *Eubacterium_fissicatena_group*, *Faecalibaculum*, *Atopostipes*, *Marvinbryantia*, *Ruminococcus*, *Christensenellaceae_R-7_group*, and *Butyricimonas* in the HFDko-wt group was significantly higher than that in the HFDwt-wt group, and the abundance of *Eubacterium_fissicatena_group* showed a significant positive correlation with LDL and vLDL levels in LF-deficient mice during lactation (Figure 5P). Therefore, the enrichment of *Eubacterium_fissicatena_group* in the HFDko-wt group may be associated with the poorer LDL and vLDL levels observed in this group.

Bivariate and multivariate correlation analysis was performed between the obesity phenotype and the gut microbial genera, and the results are shown in Figure 5P and Tables S2 and S3. In mice with normal LF intake during lactation, six bacterial taxa, including, e.g., *Bifidobacterium*, *Turicibacter*, *Eubacterium_xylanophilum_group*, etc., showed negative correlations with body weight; only *Colidextribacter* exhibited a positive correlation (Figure 5P, Table S2). The abundance of *Turicibacter* was significantly negatively correlated with serum TC levels; the abundance of *Akkermansia* was significantly positively correlated with TG levels, and the abundance of *Bifidobacterium*, *Turicibacter*, *Eubacterium_xylanophilum_group*, *Monoglobus*, and *Muribaculum* was significantly negatively correlated with LDL levels. The abundance of *Rikenella* was significantly positively correlated with serum LDL and vLDL levels. In LF-deficient mice during lactation, the abundance of *Eubacterium_xylanophilum_group*, *Monoglobus*, and *Lachnospiraceae_UCG-006* was significantly negatively correlated with serum TC levels, whereas the abundance of *Alistipes*, *Parabacteroides*, *Odoribacter*, *Bilophila*, *Rikenella*, and *A2* was significantly positively correlated with serum TC levels. The abundance of *Alistipes*, *Alloprevotella*, *Mucispirillum*, *Peptococcus*, *Tuzzerella*, and *A2* was significantly positively correlated with TG levels, and the abundance of *Alistipes*, *Parabacteroides*, *Colidextribacter*, *Odoribacter*, *Bilophila*, *Rikenella*, and *A2* was significantly positively correlated with HDL levels, while the abundance of *Eubacterium_xylanophilum_group*, *Monoglobus*, and *Lachnospiraceae_UCG-006* was significantly negatively correlated with HDL levels. The abundance of *Eubacterium_fissicatena_group* and *Streptococcus* was significantly positively correlated with LDL levels, while the abundancesof nine bacterial taxa (*Turicibacter*, *Eubacterium_xylanophilum_group*, *Monoglobus*, etc.) was significantly negatively correlated with LDL levels. Furthermore, the abundance of four bacterial taxa (*Eubacterium_fissicatena_group*, *GCA-900066575*, *Peptococcus*, and *Acetatifactor*) was significantly positively correlated with vLDL levels, whereas the abundance of another four bacterial taxa (*Monoglobus*, *Clostridium_sensu_stricto_1*, *Enterorhabdus*, and *Lachnospiraceae_UCG-006*) was significantly negatively correlated with vLDL levels. In addition, a multivariate analysis showed that the abundance of *Blautia* and *NK4A214_group* was significantly negatively associated with TG levels and positively associated with vLDL levels (Table S3). In summary, increased gut microbiota significantly correlated with serum lipoprotein levels in LF-deficient mice during lactation.

### 3.5. Alterations in the Gut Microbiota of Mice with Acute Colitis Induced by DSS

We subjected both lactating LF-intake male and female mice and LF-deficient male and female mice to acute colitis treatment and found that DSS significantly decreased the body weight and colon length of the four groups of mice (Figure S2A,B). It significantly reduced the crypt depth in male and female mice with a normal LF intake during lactation, whereas there was a downward trend in the crypt depth of mice with LF deficiency during lactation, but no significant difference was observed (Figure S2C). After DSS-induced colitis injury, LF-deficient mice showed more severe weight loss (in males), an increased DAI score (in females and males), an increased mortality rate (in males), colon shortening (in females), and increased levels of inflammatory cytokines interleukin (IL)-1β, IL-10 (in females) (published results) [9]. In this study, we compared the intestinal microbes of mice that had been induced with DSS to those that did not undergo this treatment to investigate the impact of colitis on the composition of intestinal microbes in mice. Additionally, we observed differences in the alterations in intestinal microbes following DSS stimulation between lactating LF-deficiency mice and those with regular LF intake during lactation.

**Figure 6:**
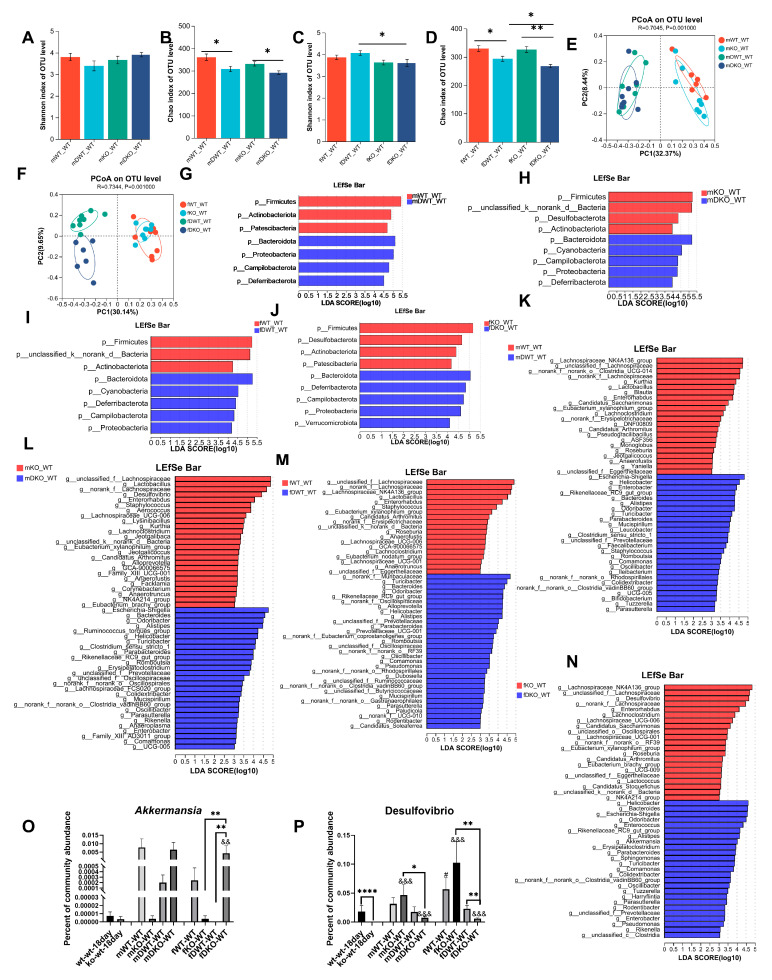
The influence of DSS on the composition of the gut microbiota in lactation LF-feeding mice and LF-deficient mice. (**A**,**B**) The impact of DSS on the gut microbiota Shannon index and Chao index in lactation LF-feeding and LF-deficient male mice. (**C**,**D**) The impact of DSS on the gut microbiota Shannon index and Chao index in lactation LF-feeding and LF-deficient female mice. (**E**) PCoA of bacterial β-diversity based on the Bray–Curtis dissimilarity index in DSS-induced colitic male model and evaluated using ANOSIM. (**F**) PCoA of bacterial β-diversity based on the Bray–Curtis dissimilarity index in DSS-induced colitic female model and evaluated using ANOSIM. (**G**) LDA scores derived from LEfSe analysis at the phylum level in the mWT-WT group and the mDWT-WT group. (**H**) LDA scores derived from LEfSe analysis at the phylum level in mKO-WT group and mDKO-WT group. (**I**) LDA scores derived from LEfSe analysis at the phylum level in fWT-WT group and the fDWT-WT group. (**J**) LDA scores derived from LEfSe analysis at the phylum level in the fKO-WT group and the fDKO-WT group. (**K**) LDA scores derived from LEfSe analysis at the genus level in the mWT-WT group and the mDWT-WT group. (**L**) LDA scores derived from LEfSe analysis at the genus level in the mKO-WT group and the mDKO-WT group. (**M**) LDA scores derived from LEfSe analysis at the genus level in the fWT-WT group and the fDWT-WT group. (**N**) LDA scores derived from LEfSe analysis at the genus level in the fKO-WT group and the fDKO-WT group. (**O**–**P**) Percentage of Akkermansia and Desulfovibrio community abundance in mice at 18 days old and 9 weeks old and in those with DSS-induced colitis. # represents a significant difference compared to 18-day-old wt-wt mice (*t*-test, # *p* < 0.05,), & represents a significant difference compared to 18-day-old ko-wt mice (*t*-test, && *p* < 0.01, &&& *p* < 0.001). (**A**–**D**,**O**,**P**) were evaluated using a two-way ANOVA. * *p* < 0.05, ** *p* < 0.01, **** *p* < 0.0001. *n* = 6–9.

In male mice, regardless of LF intake during lactation, DSS treatment had no significant effect on the Shannon index (Figure 6A) but significantly reduced the Chao index of intestinal microorganisms (Figure 6B). Similarly, in female mice, DSS treatment had no significant effect on the Shannon index (Figure 6C), but it significantly reduced the Chao index in both lactating LF-intake and LF-lack mice (Figure 6D). Furthermore, the Shannon, Chao, and Sob indices were significantly lower in the fDKO-WT group than in the fDWT-WT group (Figure 6C,D and Figure S3B). The above results indicated that DSS treatment had no effect on community diversity but could reduce the community richness of intestinal microorganisms in mice, regardless of sex and whether LF was involved in lactation. LF deficiency aggravates the decrease in α diversity caused by DSS. As shown in Figure 6E, the composition of the intestinal microflora in the mDWT-WT and mDKO-WT groups was significantly different from those in the mWT-WT and mKO-WT groups. Similarly, in female mice, the composition of the intestinal microflora in the fDWT-WT and fDKO-WT groups was significantly different from those in the fWT-WT and fKO-WT groups. These results indicated that the composition of the intestinal microflora in adult mice changed significantly after DSS treatment. Moreover, the intestinal microflora composition in LF-intake colitic mice was markedly different from that in LF-deficient colitic mice, irrespective of sex (Figure S3C).

Mice with DSS-induced acute colitis were categorized into four groups based on sex and LF intake during lactation. Each group was compared to non-colitis-induced mice at the phylum level to examine the influence of DSS enteritis on the composition of the intestinal microbiota. As shown in Figure 6G, male mice with normal LF intake during lactation experienced a significant reduction in the abundance of Firmicutes, Actinobacteria, and Patescibacteria after DSS treatment, whereas there was an increase in Bacteroidetes, Proteobacteria, Campylobacterota, and Deferribacterota. As shown in Figure 6H, male mice with LF deficiency during lactation exhibited decreased levels of Firmicutes, Desulfobacterota, and Actinobacteria after DSS treatment and increased levels of Bacteroidetes, Cyanobacteria, Campylobacterota, Proteobacteria, and Deferribacterota. In female mice with normal LF intake during lactation, DSS administration markedly reduced Firmicutes and Actinobacteria populations while enhancing Bacteroidetes, Cyanobacteria, Deferribacterota, Campylobacterota, and Proteobacteria prevalence (Figure 6I). As shown in Figure 6J, DSS administration significantly reduced Firmicutes, Desulfobacterota, Actinobacteria, and Patescibacteria and significantly increased Bacteroidetes, Deferribacterota, Campilobacterota, Proteobacteria, and Verrucomicrobiota levels in female mice with LF deficiency during lactation.

Comparisons at the genus level were performed to explore the influence of the DSS colitis model on the composition of the gut microbiota in mice (published results) [9]. In male mice with normal LF intake during lactation, the relative abundances of 22 genera such as *Kurthia*, *Lactobacillus*, *Blautia*, *Enterorhabdus*, etc., were significantly reduced in the mDWT-WT group, while the relative abundances of 26 genera, including *Escherichia-Shigella*, *Helicobacter*, *Enterobacter*, *Bacteroides*, *Alistipes*, etc., were significantly increased in the mDWT-WT group (Figure 6K). In male mice lacking LF supplementation during lactation, the relative abundances of 25 genera such as *Lactobacillus*, *Desulfovibrio*, *Enterorhabdus*, *Staphylococcus*, etc. were significantly reduced in the mDKO-WT group, while the relative abundances of 27 genera including *Escherichia-Shigella*, *Bacteroides*, *Odoribacter*, *Alistipes*, etc. were significantly increased in the mDKO-WT group (Figure 6L). In female mice with normal LF intake during lactation, DSS treatment decreased the abundance of 19 genera, including *Lachnospiraceae_NK4A136_group*, *Lactobacillus*, *Enterorhabdus*, *Staphylococcus*, etc., and increased the abundance of 31 genera, including *Turicibacter*, *Bacteroides*, *Odoribacter*, *Alloprevotella*, *Helicobacter*, and *Alistipes*, among others (Figure 6M). In female mice lacking LF supplementation during lactation, DSS colitis treatment decreased the abundance of 21 genera including *Lachnospiraceae_NK4A136_group*, *Desulfovibrio*, *Enterorhabdus*, etc., and increased the abundance of 25 genera including *Helicobacter*, *Bacteroides*, *Escherichia-Shigella*, *Odoribacter*, among others (Figure 6N).

### 3.6. Effects of LF Deficiency During Lactation on Intestinal Microbial Composition in CUMS-Induced Depression-like Behavior Mice

After four weeks of CUMS modeling, lactating LF-deficient male mice exhibited significantly lower sucrose preference and increased immobility time in the forced swimming test (FST) and the tail suspension test (TST) compared to before modeling, indicating successful modeling of depression. Compared with LF-intake mice during lactation, 4-week CUMS induced more severe depression-like behaviors in LF-deficient mice, such as low sucrose preference, more FST immobility time, and less central area distance in the open field test. In terms of serological indices, 4 weeks of CUMS resulted in LF-deficient mice exhibiting lower BDNF and higher CORT, ACTH, TNF-α, IL-1β, and LPS than LF-intake mice (published results) [9].

**Figure 7:**
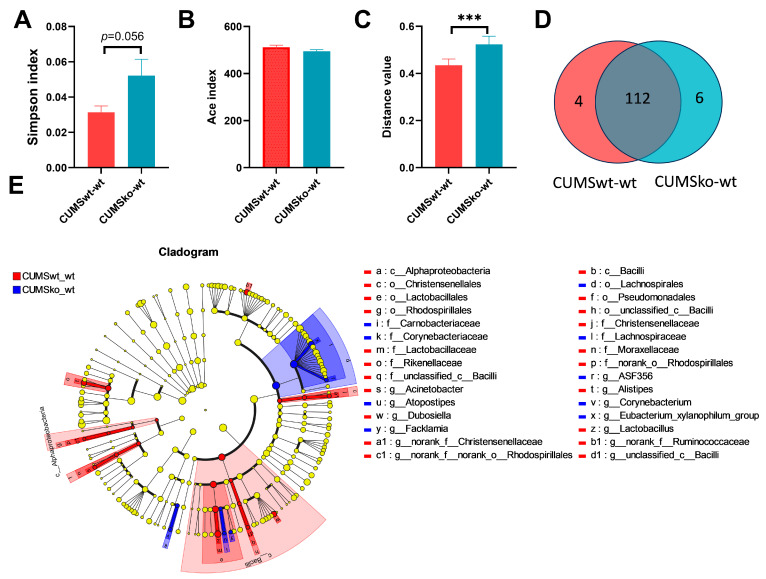
Effects of LF during lactation on intestinal microbial composition of CUMS-induced depression model. (**A**) Simpson index. (**B**) Ace index difference analysis between LF-intake and LF-deficient mice in CUMS-induced depression model. (**C**) β diversity difference analysis between LF-intake and LF-deficient mice in CUMS-induced depression model. (**D**) Venn diagram analysis between CUMSwt_wt and CUMSko_wt at the genus level. (**E**) LEfSe comparison between CUMSwt_wt and CUMSko_wt mice from the phylum level to the genus level. LDA > 3. (**A**–**C**) were evaluated using Student’s-*t*-test, *** *p* < 0.001. *n* = 8.

Comparison of the intestinal microbiota of CUMSwt-wt mice with that of CUMSko-wt mice revealed that there was no significant difference in α-diversity of the intestinal microbiota between the two groups. The Simpson index of the CUMSko-wt mice was higher than that of the CUMSwt-wt mice (*p* = 0.056), indicating that the community diversity of the CUMSko-wt mice was lower than that of the CUMSwt-wt group (Figure 7A,B). β-diversity differences analysis showed a significant difference between the intestinal microbiota of the two groups of mice (Figure 7C). The Venn diagram illustrates that there were 122 common taxa at the genus level between the two groups of mice, with four genera (*Anaerovorax*, *unclassified_f_Christensenellaceae*, *Ruminiclostridium*, *Acinetobacter*) specifically enriched in CUMSwt-wt mice and six genera (*Mucispirillum*, *unclassified_f_Erysipelotrichaceae*, *Facklamia*, *Candidatus_Soleaferrea*, *Corynebacterium*, *Atopostipes*) specifically enriched in CUMSko-wt mice (Figure 7D). A Lefse discriminant analysis revealed no differential phyla between the two groups, but nine families and 13 genera showed differences (Figure 7E).

### 3.7. The Connections Between Bacteria That Are Enriched in Various Models

We compared the alterations in bacterial genera associated with aging, those induced via HFD, and those induced via DSS, and we compiled the selected genera exhibiting specific regularities in Table S4. First, the bacteria that showed the same pattern of change in male DSS-induced colitis mice and HFD-induced obese mice were compared (Table 1). In mice with normal LF intake during lactation, both DSS and HFD significantly increased the abundance of *Alistipes*, *Colidextribacter*, *Mucispirillum*, *Odoribacter*, and *Oscillibacter* and significantly decreased the abundance of *Monoglobus*, *Muribaculum*, and *Eubacterium_xylanophilum_group.* In LF feeding-deficient mice during lactation, both DSS and HFD significantly reduced the abundance of *Eubacterium_xylanophilum_group*, *Lachnoclostridium*, *Lachnospiraceae_UCG-006*, and *Staphylococcus*, but there was no similar increase in bacterial genera.

Furthermore, several bacterial taxa exhibited divergent responses in colitis versus obesity models. In male mice with a normal LF intake during lactation, DSS treatment significantly increased the abundance of *Clostridium_sensu_stricto_1* and *Turicibacter*, and significantly decreased the abundance of *Ruminococcaceae_NK4A214_group* and *Roseburia*. Conversely, HFD exposure showed inverse effects. In lactating LF-deficient mice, DSS treatment increased the abundance of *Clostridium_sensu_stricto_1* and *Turicibacter*, decreased the abundance of *Anaerotruncus*, *GCA-900066575*, *Ruminococcaceae_NK4A214_group*, and *Roseburia*, and HFD caused the opposite trend of these bacteria.

Comparing changes in the gut microbiome with age to changes in the gut microbiome caused by DSS (Table 2), we found that in male mice with normal LF intake during lactation, with increasing age, the abundances of *Candidatus_Arthromitus*, *Candidatus_Saccharimonas*, *Enterorhabdus*, *Eubacterium_xylanophilum_group*, *Jeotgalicoccus*, *Kurthia*, and *Monoglobus* increased significantly, and the abundances of *Bacteroides Mucispirillum*, *Oscillibacter*, *Parabacteroides*, *Romboutsia*, and *Turicibacter* decreased significantly. However, DSS treatment resulted in the opposite trend in the abundance of these bacteria. In lactating LF-deficient male mice, with increasing age, the abundance of 13 genera (*Aerococcus*, *Candidatus_Arthromitus*, *Corynebacterium*, *Desulfovibrio*, *Enterorhabdus*, *Eubacterium_brachy_group*, *Lactobacillus*, etc.) significantly increases. However, enteritis decreased the abundance of these genera. Conversely, with increasing age, the abundance of *Bacteroides*, *Oscillibacter*, *Colidextribacter*, *Odoribacter*, and *Rikenella* significantly decreased; however, enteritis caused an increase in their abundance. In lactation-LF-intake female mice, the abundance of *Candidatus_Arthromitus*, *Enterorhabdus*, *Eubacterium_xylanophilum_group*, *Lachnospiraceae_UCG-006*, *Lactobacillus*, and *Staphylococcus* significantly increased with age, while the abundance of *Mucispirillum*, *Oscillibacter*, *Parabacteroides*, and *Turicibacter* significantly decreased. However, DSS-induced colitis led to an opposite trend in the abundance of these bacteria. In lactating LF-deficient female mice, the abundance of *Candidatus_Arthromitus*, *Candidatus_Saccharimonas*, *Enterorhabdus*, *Desulfovibrio*, *Eubacterium_brachy_group*, and *Lachnoclostridium* increased with age, while the abundance of *Bacteroides*, *Oscillibacter*, *Colidextribacter*, and *Odoribacter* decreased. Similarly, DSS-induced colitis led to the opposite trend in the abundance of these bacteria. Under physiological conditions, the stability of the intestinal microbiota in adult mice is higher than that in lactating mice; thus, age-related changes in the intestinal microbiota may be more conducive to maintaining stability. Conversely, colitis causes a reversal in these changes, which is a significant factor contributing to the reduced stability of the intestinal microbiota.

Changes in the gut microbiota with age were compared with the changes induced via HFD (Table 3). In male mice with normal LF intake during lactation, the abundance of *Eubacterium_xylanophilum_group* and *Monoglobus* significantly increased with age, but HFD led to a significant decrease in these bacteria. As age increased, the abundances of *Anaerotruncus*, *Bilophila*, *Blautia*, *Mucispirillum*, *Oscillibacter*, *Rikenella*, and *Roseburia* significantly decreased, but they significantly increased in the HFD group. However, the abundances of *Muribaculum* and *Turicibacter* decreased in both the HFD and age models. In male mice with LF deficiency during lactation, the abundance of *Dubosiella*, *Lachnospiraceae_UCG-006*, and *Staphylococcus* significantly increased with age, while the abundance of *Anaerotruncus*, *Roseburia*, and *GCA-900066575* significantly decreased; however, opposite trends of these bacteria were observed under HFD stimulation, and no genera exhibited similar trends in either the HFD or age models.

Table S5 summarizes the genera showing similar trends in different disease models affected by LF deficiency during lactation. Compared to mice with normal LF intake during lactation, LF-deficient mice showed a significant increase in the abundance of Marvinbryantia in HFD-induced obesity and DSS-induced colitis models. Furthermore, LF-deficient mice showed a significant increase in the abundance of Atopostipes and a decrease in the abundance of Alistipes, Parasutterella, and Rikenella in HFD-induced obesity and CUMS-induced depression models compared with LF-feeding mice.

## 4. Discussion

Microbiota are found on all body surfaces exposed to the environment, including the skin, mouth, lungs, reproductive system, and urinary tract. The greatest density and diversity of microbes are found in the gastrointestinal tract. Gut microbes play an important role in the homeostasis of host physiological systems such as the brain, intestine, and pancreas [10]. A metagenomic analysis revealed that the gut microbiota composition is transformed throughout the early stages of human development and is influenced by diet. The infant gut microbiota undergoes substantial development during the first three years of life, marked by progressive increases in diversity and stabilization [11]. By 3–5 years of age, the gut microbiota reaches a relatively stable state, resembling adult-like composition, potentially driven by dietary convergence [12]. Our study investigated the effect of LF deficiency during lactation on changes in the gut microbiota composition of mice from lactation to adulthood. Wu et al. found that the α diversity of male mice showed significant age-dependent increases from 3 months to 28 months [13]. In contrast to previous research findings, there was no significant change in α-diversity between 9-week-old and 18-day-old mice with normal LF intake (Figure 2A–D). We speculate that this may be related to the different age groups and shorter time intervals. During the lactation period, a decrease in the diversity of adult mouse microbiota was observed in LF-deficient mice (Figure 3B), indicating the persistent and negative impact of LF deficiency during the lactation period on the establishment of gut microbiota homeostasis. This may also be related to the marked reduction in the α-diversity of gut microbiota in LF-deficient mice after HFD modeling and DSS colitis treatment (Figure 5A–D and Figure 6C,D), suggesting that the establishment of early-life gut microbiota lays the foundation for lifelong microbial community and has far-reaching implications for the development of late-life diseases [14]. Dietary lipids serve as substrates for intestinal bacterial metabolism and can also inhibit bacterial growth through toxic effects [15]. Consistent with our research findings, numerous studies have demonstrated that HFD can lead to a decrease in gut microbiota diversity, but some studies have found an increase in gut microbiota diversity in mice fed an HFD, which is related to the content of specific nutrients added to the diet [16,17]. Similar to our findings, DSS-induced acute colitis exhibited a significant decrease in α-diversity [18].

β diversity analysis revealed that age, HFD, and DSS treatment each significantly influenced gut microbial community structure. Table 4 summarizes the effects of the different influencing factors at the phylum level. Firmicutes and Bacteroidetes constitute the two predominant phyla in the gut microbiota, collectively accounting for more than 90% of the total bacterial population [19]. In our study, compared with 18-day-old mice, the abundance of Firmicutes increased, and that of Bacteroidetes decreased in the gut microbiota of adult mice. Similar findings have been observed in the human gut microbiota, i.e., the F/B ratio evolved during different life stages. For infants, adults, and elderly individuals, the measured ratios are 0.4, 10.9, and 0.6, respectively [20]. The abundance of Firmicutes is related to the ability to harvest energy from food, suggesting that adult mice have an increased ability to extract energy compared with lactating mice. The F/B ratio has frequently been considered a possible hallmark of obesity. Our study found that HFD-fed LF-deficient mice showed a significantly increased F/B ratio, indicating a more severe obesity disorder in LF-deficient mice. However, the relationship between the F/B ratio and obesity has been challenged, as many studies have found no change or even a decrease in the F/B ratio in obesity models [19]. Meta-analyses of human gut microbiota and obesity have also not found a clear trend between the F/B ratio and obesity status [21]. Similarly, in our experimental results, the F/B ratio in the HFDwt-wt group did not change significantly despite a significant increase in body weight and serum TC, HDL, and LDL levels (unpublished results). Therefore, the complexity of how the gut microbiota regulates obesity far exceeds the simple imbalance between symbiotic phyla. In the DSS-induced colitis model, regardless of whether the mice were male or female and whether they were lactating or consuming LF, DSS treatment significantly decreased the abundance of Firmicutes and Actinobacteria and increased the abundance of Bacteroidetes, Campylobacterota, Deferribacterota, and Proteobacteria. In clinical practice, the overgrowth of Proteobacteria is a characteristic of dysbiosis in patients with IBD [22]. The increase in Proteobacteria in DSS mice was similar to that observed in patients with IBD. Notably, DSS treatment reduces the abundance of Desulfobacterota in LF-deficient mice; however, DSS increases the abundance of Desulfobacterota [23].

The β-diversity results indicate significant differences in the gut microbiota of LF-intake and LF-deficient mice across various groups, including 18-day-old mice, adult healthy mice, the HFD-induced obesity model, DSS-induced colitic model, and CUMS-induced depression model (Figure 4C, Figure 5F, Figure 7C and Figure S3C). In 18-day-old mice, *Oscillibacter*, *Bilophila*, *Colidextribacter*, *Harryfintia*, and *Odoribacter*, which are associated with inflammation, were enriched in the ko-wt group [9] (Figure 4D). In adult female mice, LF deficiency increased the abundance of the potentially pathogenic microbe *Dubosiella* and decreased that of the beneficial bacterium *Eubacterium_nodatum_group* [9] (Figure 4F). In an obesity model, the abundances of the beneficial bacteria *Blautia* [24] and short-chain fatty acid (SCFA)-producing bacteria *Alloprevotella* [25] and *Alistipes* [26] were significantly higher in the HFDwt-wt group than in the HFDko-wt group. In mice lacking LF during lactation, *Ruminococcus* abundance increased significantly after HFD treatment compared to HFDwt-wt mice, potentially exacerbating obesity through pro-inflammatory mechanisms [27]. Consequently, the absence of LF during lactation exacerbates gut microbiota dysbiosis induced via the HFD, which may be related to the worse serum cholesterol index of the HFDko-wt group. Similarly, our previous research indicated that the absence of LF during lactation exacerbates the dysbiosis of the intestinal microbiota induced via DSS and CUMS [9]. Further analyses revealed that LF deficiency during lactation may lead to functional alterations in certain microorganisms. *Enterorhabdus* abundance was positively correlated with vLDL levels in mice with normal LF intake, whereas there was a significant negative correlation in LF-deficient mice. Other microbial genera that exhibited opposite correlations between the two feeding conditions included *GCA-900066575*, *Peptococcus*, and *Tuzzerella* (Figure 5P, r > 0.3)*. Akkermansia*, representing the phylum Verrucomicrobiota, is a beneficial genus, with decreased abundance observed in the DSS-induced colitis model [28]. However, similar studies have shown an increase in *Akkermansia* abundance in DSS-induced colitis models [18]. Notably, in our study, the DSS-induced increase in *Akkermansia* abundance was evident only in LF-deficient mice. Conversely, in mice with normal LF intake, DSS led to the downregulation of *Akkermansia* colonization (Figure 5O). Therefore, the impact of LF deficiency during the lactation period on the intestinal environment raises the question of why DSS increases *Akkermansia* abundance, and whether the functionality of *Akkermansia* has undergone alterations warrants further investigation. Our results indicated a significantly lower abundance of *Desulfovibrio* in the ko-wt group than in the wt-wt group in 18-day-old mice. Furthermore, *Desulfovibrio* abundance increased notably with age in ko-wt mice, followed by a significant decrease after DSS stimulation (Figure 6P). In contrast, DSS stimulation did not significantly change *Desulfovibrio* abundance in the wt-wt group (Figure 6P). These findings suggest that LF intake during lactation is beneficial for the early colonization of *Desulfovibrio*, which may contribute to the stability of this microorganism. *Desulfovibrio*, a prominent representative sulfate-reducing bacterium in the gut microbiota known for its capacity to generate H2S, is enriched in patients with IBD [29,30]. In contrast to previous reports, our study demonstrated a decrease in *Desulfovibrio* abundance following DSS administration in LF-deficient mice. The altered effect of *Desulfovibrio* on the host in an LF-deficient gut environment requires further investigation.

Clinical evidence indicates a strong correlation between chronic HFD intake and both IBD progression and clinical relapse [31]. Investigating the shared changes in gut microbiota between mice with HFD and colitis could provide insights into the microbial perspective of the relationship between IBD and obesity and provide a novel therapeutic approach and theoretical framework for modulating gut microbiota in IBD patients with prolonged HFD exposure. We found that both DSS and HFD significantly elevated the levels of *Alistipes*, *Colidextribacter*, *Mucispirillum*, *Odoribacter*, and *Oscillibacter*. *Alistipes*, a recently identified bacterial genus, exhibits context-dependent roles in gut health, functioning as both a beneficial commensal and a conditional pathogen [26]. *Alistipes* abundance was increased through HFD [32], and it participates in many putrefaction pathways, producing harmful metabolites and damaging colonic cells [26]. *Alistipes* promote Colorectal cancer (CRC) through the IL-6/STAT 3 pathway, suggesting its potential as a CRC biomarker. The abundance of *Colidextribacter* has been reported to be negatively correlated with antioxidant capacity [33]. *Mucispirillum* and *Odoribacter* have been reported to be pro-inflammatory bacteria [34]. Based on the performance of microorganisms, inflammation, and oxidative stress are important mechanisms linking HFD to IBD. HFD activates the TLR/NF-κB and oxidative stress pathways, thereby bolstering intestinal inflammation [35]. Among the genera that were concomitantly reduced in the HFD and DSS models, *Muribaculum* was the first described genus belonging to *Muribaculaceae* family [36]. *Muribaculaceae* showed an inverse correlation with inflammatory markers but demonstrated direct proportionality to intestinal barrier components [37]. Similarly to our findings, the abundance of *Muribaculum* was reduced in both the colitis and HFD mouse models [18,38]. Our findings revealed a decrease in the number of bacterial genera that changed in both HFD and colitis models in LF-deficient mice. We speculate that the association between HFD and IBD may be weakened or may undergo other changes in lactating LF-deficient mice. Therefore, relevant experiments must be designed to address this issue. Consistent with our findings, the abundance of *Clostridium_sensu_stricto_1* [39,40] and *Turicibacter* [18,41] increased in colitis and decreased in the HFD-induced obesity model. The prevalence of *Clostridium_sensu_stricto_1* is linked to an increased presence of the metabolite cinnamyl alcohol [40]. This compound impedes the generation of new fat cells and enhances fat breakdown through the PPAR signaling pathway [40]. *Turicibacter* strains can alter host bile acid and lipid metabolism and are often negatively correlated with dietary fat and host adiposity [42]; however, they have been reported as harmful bacteria in colitis models [43]. Contrary to our research findings, *Roseburia* [44] and *Ruminococcaceae_NK4A214_group* [18,45] are beneficial bacteria and show a decreased abundance in both colitis and HFD models.

Our findings revealed that the effect of DSS on several microbial phyla in the gut was opposite to that of the age model. For instance, Firmicutes, Bacteroidetes, Actinobacteria, Deferribacterota, and Campylobacterota exhibited contrasting trends between the DSS and age models, with only Cyanobacteria showing a consistent pattern of change in both the DSS and age models (Table 4). At the genus level, numerous bacteria exhibited opposing trends, indicating a significant influence of DSS on intestinal microbiota dysbiosis. An imbalance in gut microbial composition is established as a key etiological factor in IBD [46]; however, intestinal microbial imbalance was similarly observed in Axl-/- mice, yet no clinical symptoms of colitis were present, indicating a lack of a causal relationship between imbalance and colitis symptoms [22]. An increase in beneficial bacterial abundance and a decrease in harmful bacterial abundance are considered the most common manifestations of dysbiosis. However, it is challenging to categorize many microbes as either beneficial or harmful because their functions can vary in different microbial environments. In our study, we found that DSS-induced gut microbiota dysbiosis exhibited a trend opposite to that of intestinal microbiota maturation. Similarly, we compared the impact of HFD on the microbiota with changes in the microbiota over time and found that, out of the five phyla that changed in HFDwt-wt mice, four exhibited opposite trends to those in the age model. However, in HFDko-wt mice, only one phylum exhibited an opposite trend to that in the age model. At the genus level, over one-third of the HFD-induced changes in the gut microbiota genera exhibited opposite trends to the changes in the gut microbiota during gut maturation. Therefore, designing strategies based on the succession of bacterial consortia during the maturation of the intestinal microbiota may offer a novel direction for ameliorating gut microbiota dysbiosis in patients with IBD and obesity.

Reducing Gram-negative bacterial abundance may facilitate the development of a mature gut microbiota. Our study reveals that Gram-negative bacteria carrying LPS decrease with age in mice, regardless of LF intake status. As LPS is a common virulence factor that compromises intestinal barrier integrity and promotes host inflammation [47], this age-related reduction may confer health benefits. In addition, the Contains-Mobile-Elements functional category increases with age in mice receiving normal LF intake, but not in LF-deficient mice. Given that enriched mobile genetic elements facilitate the interspecies transfer of functional traits, accelerating bacterial evolution and environmental adaptation [48], this divergence may explain why LF-deficient mice exhibit more pronounced microbial dysbiosis under pathological conditions.

This study involved several limitations. (1) Model system limitation: We acknowledge that our findings are derived from a mouse model. While mouse models provide mechanistic insights, fundamental differences exist between mouse and human gut microbiome development, immune function, and LF metabolism. The direct extrapolation of the observed microbiota effects to human development requires caution. (2) Controlled intervention vs. human complexity: Experimental challenges (HFD/DSS/CUMS) were administered under standardized conditions. Human disease involves multifactorial interactions (genetics, diet complexity, and comorbidities) not captured here. (3) Microbiome functional inference: the observed bacterial signatures may manifest differently in heterogeneous human populations.

## 5. Conclusions

This study determined the changes in the gut microbiome in mice with normal LF intake during lactation and in LF-deficient mice using age, HFD, and enteritis models. Differences in the gut microbiome changes between the two feeding conditions in the obesity, IBD, and depression models were compared. We found that LF deficiency during lactation increased the abundance of potentially pathogenic microbiota in 18-day-old mice, and the abundance of potentially pathogenic microbiota decreased significantly with age, although it remained significantly higher than in mice with a normal LF intake during lactation. When challenged with HFD, DSS, or CUMS, LF-deficient mice showed more severe gut microbiome dysbiosis, and LF deficiency during lactation may alter the functions of *Enterorhabdus*, *GCA-900066575*, *Peptococcus*, *Tuzzerella*, *Akkermansia*, and *Desulfovibrio*. Furthermore, a comparison of the gut microbiota changes in different models revealed that the bacteria upregulated via both HFD and DSS exhibited pro-inflammatory and pro-oxidative functional characteristics. During lactation, LF-deficient mice showed a decrease in the number of genera with similar changing trends in both HFD- and DSS-induced colitis models, suggesting that LF deficiency during lactation may weaken the connection between HFD and IBD. Many changes in the gut microbiota induced via DSS and HFD showed opposite trends to those observed with aging. Regulating the gut microbiota according to its regular succession of gut microbiota during the process of gut microbial maturation may be beneficial in ameliorating gut microbiome dysbiosis in obese and IBD patients.

## Figures and Tables

**Table 1:** Comparison of gut microbiota between HFD and DSS models.

Genus	HFD		DSS	
	wt-wt	ko-wt	wt-wt	ko-wt
*Alistipes*	↑	-	↑ ↑	↑ ↑
*Colidextribacter*	↑	-	↑ -	↑ ↑
*Mucispirillum*	↑	-	↑ ↑	↑ ↑
*Odoribacter*	↑	-	↑ ↑	↑ ↑
*Oscillibacter*	↑	-	↑ ↑	↑ ↑
*Monoglobus*	↓	-	↓ -	- -
*Muribaculum*	↓	-	↓ -	↓ ↓
*Eubacterium_xylanophilum_group*	↓	↓	↓ ↓	↓ ↓
*Lachnoclostridium*	-	↓	↓ ↓	↓ ↓
*Lachnospiraceae_UCG-06*	-	↓	- ↓	↓ -
*Staphylococcus*	-	↓	↑ ↓	↓ -
*Clostridium_sensu_stricto_1*	↓	↓	↑ -	↑ -
*Turicibacter*	↓	↓	↑ ↑	↑ ↑
*NK4A214_group*	↑	↑	↓ -	↓ ↓
*Roseburia*	↑	↑	↓ ↓	↓ ↓
*GCA-900066575*	-	↑	- ↓	↓ -
*Anaerotruncus*	↑	↑	- ↓	↓ ↓

“-”, no change; “↑”, upregulate; “↓”, downregulate. Red arrow represents female mice; blue represents male mice.

**Table 2:** Comparison of gut microbiota between Aging and DSS models.

Genus	Age		DSS	
	wt-wt	ko-wt	wt-wt	ko-wt
*Candidatus_Arthromitus*	↑ ↑	↑ ↑	↓ ↓	↓ ↓
*Candidatus_Saccharimonas*	↑ ↑	↑ ↑	↓ -	- ↓
*Enterorhabdus*	↑ ↑	↑ ↑	↓ ↓	↓ ↓
*Eubacterium_xylanophilum_group*	↑ ↑	- -	↓ ↓	↓ ↓
*Jeotgalicoccus*	↑ -	↑ -	↓ ↓	↓ -
*Kurthia*	↑ -	↑ -	↓ -	↓ -
*Monoglobus*	↑ ↑	- -	↓ -	- -
*Bacteroides*	↓ ↓	↓ ↓	↑ -	↑ ↑
*Mucispirillum*	↓ ↓	- -	↑ ↑	↑ ↑
*Oscillibacter*	↓ ↓	↓ ↓	↑ ↑	↑ ↑
*Parabacteroides*	↓ ↓	- -	↑ ↑	↑ ↑
*Romboutsia*	↓ -	- -	↑ ↑	↑ -
*Turicibacter*	↓ ↓	- -	↑ ↑	↑ ↑
*Aerococcus*	- -	↑ -	↓ -	↓ -
*Desulfovibrio*	- ↑	↑ ↑	- -	↓ ↓
*Eubacterium_brachy_group*	- -	↑ ↑	↓ ↓	↓ ↓
*Corynebacterium*	- -	↑ -	- -	↓ -
*Lachnospiraceae_UCG-06*	- ↑	↑ -	- ↓	↓ -
*Lactobacillus*	- ↑	↑ ↑	↓ ↓	↓ -
*Lysinibacillus*	- -	↑ -	- -	↓ -
*Microbacterium*	- -	↑ -	- -	↓ -
*Staphylococcus*	↑ ↑	↑ -	↑ ↓	↓ -
*Colidextribacter*	- -	↓ ↓	↑ -	↑ ↑
*Odoribacter*	- -	↓ ↓	↑ ↑	↑ ↑
*Rikenella*	↓ -	↓ -	- -	↑ ↑
*Lachnoclostridium*	- -	- ↑	↓ ↓	↓ ↓

“-”, no change; “↑”, upregulate; “↓”, downregulate. Red arrow represents female mice; blue represents male mice.

**Table 3:** Comparison of gut microbiota between Aging and HFD models.

Genus	Age		HFD	
	wt-wt	ko-wt	wt-wt	ko-wt
*Eubacterium_xylanophilum_group*	↑ ↑	- -	↓	↓
*Monoglobus*	↑ ↑	- -	↓	-
*Anaerotruncus*	↓ ↓	↓ ↓	↑	↑
*Bilophila*	↓ ↓	↓ ↓	↑	-
*Blautia*	↓ ↓	- -	↑	-
*Mucispirillum*	↓ ↓	- -	↑	-
*Oscillibacter*	↓ ↓	↓ ↓	↑	-
*Rikenella*	↓ -	↓ -	↑	-
*Roseburia*	↓ -	↓ -	↑	↑
*Muribaculum*	↓ ↓	↓ ↓	↓	-
*Turicibacter*	↓ ↓	- -	↓	↓
*Dubosiella*	- -	↑ ↑	↓	↓
*Lachnospiraceae_UCG-06*	- ↑	↑ -	-	↓
*Staphylococcus*	↑ ↑	↑ -	-	↓
*GCA-900066575*	↓ ↓	↓ ↓	-	↑

“-”, no change; “↑”, up regulate; “↓”, down regulate. Red arrow represents female mice; blue represents male mice.

**Table 4:** The summary of gut microbiota at the phylum level in three models.

	Age		HFD		DSS	
	wt-wt	ko-wt	wt-wt	ko-wt	wt-wt	ko-wt
Firmicutes	↑	↑	-	↑	↓	↓
Bacteroidota	↓	↓	-	↓	↑	↑
Actinobacteriota	↑	↑	↓	↓	↓	↓
Cyanobacteria	↑	↑	↓	-	↑	↑
Deferribacterota	↓	↓	↑	-	↑	↑
Campilobacterota	↓	↓	↑	-	↑	↑
Patescibacteria	↑	↑	-	-	-	↓
Acidobacteriota	↓	↓	-	-	-	-
Desulfobacterota	-	-	-	-	-	↓
Proteobacteria	-	-	-	-	↑	↑
Verrucomicrobiota	-	-	↑	↑	-	↑

LDA > 3, the red arrow represents only female mice, the blue represents only male mice, and the black represents both female and male mice. “-”, no change; “↑”, upregulate; “↓”, downregulate.

## Data Availability

All relevant data are included in the main manuscript or Supplementary Files. Raw sequence data are available in the NCBI Short Read Archive with the following project IDs: PRJNA1030344, PRJNA1030310, and PRJNA1256538.

## Supplementary Materials

The following supporting information can be downloaded at: https://www.mdpi.com/article/10.3390/nu17132248/s1, Figure S1: The bacterial phenotypes based on BugBase; Figure S2: The damage caused by DSS in mice; Figure S3: Effects of lactoferrin on α and β diversity of microflora in mice with colitis; Table S1: Chronic unpredictable mild stress schedule; Table S2: Multivariate analysis results in LF-feeding mice; Table S3: Multivariate analysis results in LF-lacking mice; Table S4: Summary of changes in bacteria genera with the same or opposite trend in the three models; Table S5: Genus with consistent impact trends on three models in the absence of LF during lactation.

## Abbreviations

IBD	inflammatory bowel disease
LF	lactoferrin
HFD	high-fat diet
DSS	dextran sodium sulfate
KO	knockout
WT	wild-type
OTU	operational taxonomic units
PCoA	principal coordinate analysis plots
TC	total cholesterol
HDL	high-density lipoprotein
LDL	low-density lipoprotein
CUMS	chronic unpredictable mild stress
CRC	colorectal cancer
SCFA	short-chain fatty acid
