# Supplementary material for: Effects of Lactation Lactoferrin Deficiency on Intestinal Microbiota in Different Mice Models
Source: Nutrients. 2025 Jul 7;17(13):2248. doi: 10.3390/nu17132248 (PMC12252203; doi:10.3390/nu17132248)
Supplement: Supplementary file 1 [file nutrients-17-02248-s001.zip › nutrients-3699895-supplementary.pdf]

Supplementary Material for

Effects of lactation lactoferrin deficiency on intestinal microbiota in different mice models

Wenli Wang[1,2], Qin An[1], Yunxia Zou[1], Qingyong Meng[3], Yali Zhang[1*]

[1]College of Food Science and Nutritional Engineering, China Agricultural University, Beijing, China

[2]The International Peace Maternity and Child Health Hospital, Shanghai Jiao Tong University School of Medicine, Shanghai, China

[3]College of Biological Sciences, China Agricultural University, Beijing, China

[*] To whom correspondence should be addressed.

Yali Zhang

e-mail: zhangyali@cau.edu.cn

Figure S1

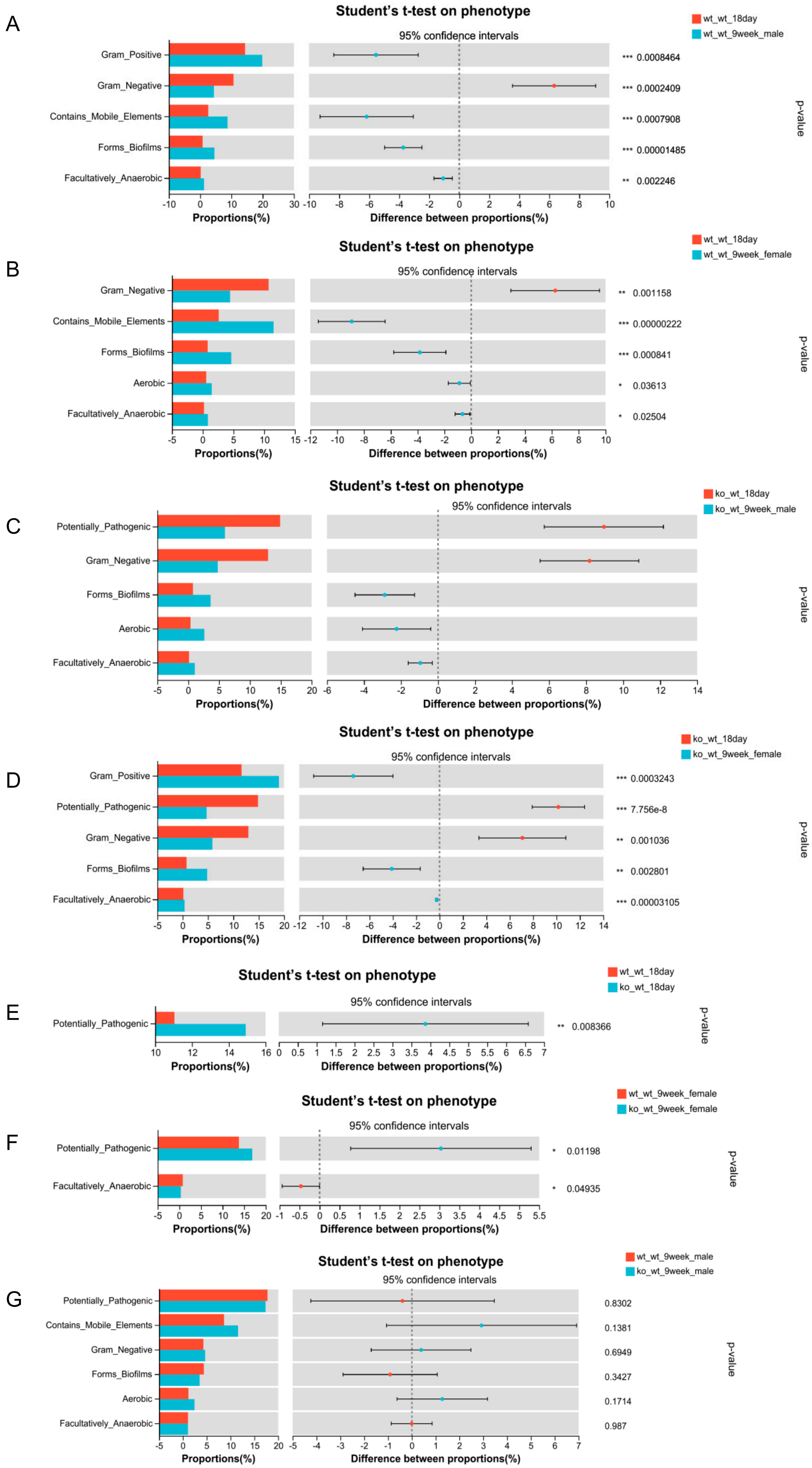
A
B
C
D
E
F
G
Student's t-test on phenotype
95% confidence intervals
Proportions(%)
Difference between proportions(%)
p-value
wt_wt_18day
wt_wt_9week_male
wt_wt_9week_female
ko_wt_18day
ko_wt_9week_male
ko_wt_9week_female
Gram_Positive
Gram_Negative
Contains_Mobile_Elements
Forms_Biofilms
Facultatively_Anaerobic
Aerobic
Potentially_Pathogenic
*** 0.0008464
*** 0.0002409
*** 0.0007908
*** 0.00001485
** 0.002246
** 0.001158
*** 0.00000222
*** 0.000841
* 0.03613
* 0.02504
*** 0.0003243
*** 7.756e-8
** 0.001036
** 0.002801
*** 0.00003105
** 0.008366
* 0.01198
* 0.04935
0.8302
0.1381
0.6949
0.3427
0.1714
0.987

Figure S1. The bacterial phenotypes based on BugBase. A. Comparison of bacterial phenotype between wt-wt-18day group and wt-wt-9week-male group. B. Comparison of bacterial phenotype between wt-wt-18day group and wt-wt-9week-female group. C. Comparison of bacterial phenotype between ko-wt-18day group and ko-wt-9week-male group. D. Comparison of bacterial phenotype between ko-wt-18day group and ko-wt-9week-female group.E. Comparison of bacterial phenotype between wt-wt-18day group and ko-wt-18day group.F. Comparison of bacterial phenotype between wt-wt-9week-female and ko-wt-9week-female group. G. Comparison of bacterial phenotype between wt-wt-9week-male and ko-wt-9week-male group. n=8-9. A-C were evaluated using Students's t-test.

Figure S2

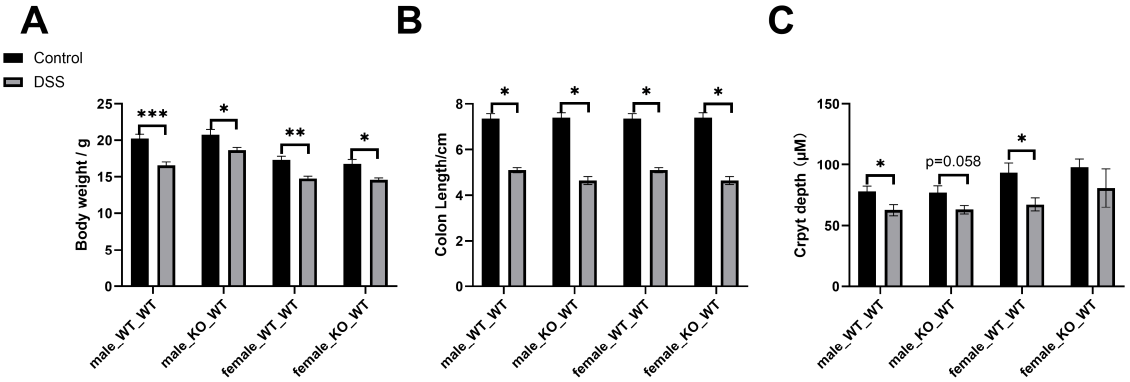

Figure S2. The damage caused by DSS in mice. A. Effect of DSS on body weight of mice. B. Effect of DSS on colon length of mice. C. Effect of DSS on crypt depth of mice. n=7-9. A-C were evaluated using Students's t-test.

Figure S3

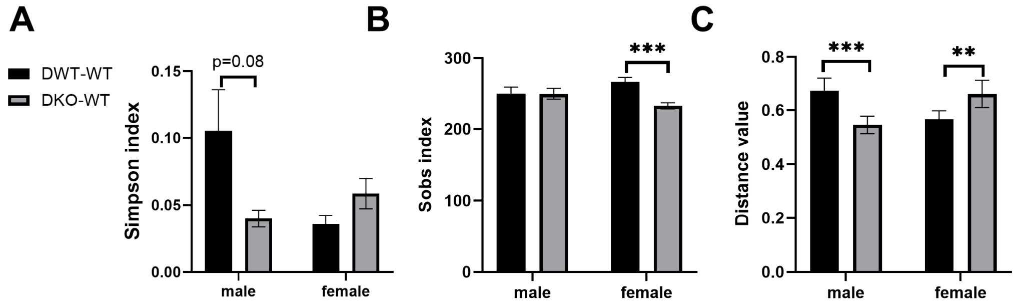

Figure S3. Effects of lactoferrin on α and β diversity of microflora in mice with colitis. A. Effect of lactoferrin on Simpson index of microflora in colitis mice. B. Effect of lactoferrin on Sob index of microflora in colitis mice. C. β diversity difference analysis in colitis mice.n=7-8. A-C were evaluated using Students's t-test.

Table S1 Chronic unpredictable mild stress schedule

Stressors	Details	Days
**Tail clamping**	Tail pinch 1cm apart from the end of the tail for 6min.	1, 10, 19, 23
**Force swimming**	Mice were placed for 6 min in a cylindrical clear plastic tank (30cm high * 10cm diameter) filled with water (23±1°C) to a depth of 20cm. Immediately after the swim, mice were removed from the tank and towel-dried before being placed back in home cages.	2, 9, 17, 28
**Food and water deprivation**	Mice were subjected to 24h of food and water deprivation. Food and water were provided immediately after the end of the fasting period.	3, 8, 21, 27
**Wet cage**	200ml water in 100g bedding for 24h. Immediately after the swim, mice were removed from the tank and towel-dried before being placed back in home cages.	4, 11, 15, 24
**Cage tilting**	Cage tilting (45°) along the vertical axis for 24h.	5, 14, 20, 25
**Restraint**	Mice were individually restraint for 4h inside 50 ml centrifuge tubes with proper holes for ventilation.	6, 12, 16, 22
**Shaking**	Mice were shaking for 15min in 150r/min	7, 13, 18, 26

Table S2 Multivariate analysis results in LF-feeding mice.

Dependent variable	BW		TG		R2	F
	B	SE	B	SE		
Turicibacter	-0.001*	0	-0.007	0.033	0.453	4.551*
Eubacterium_xylanophilum_group	-0.001*	0	-0.01	0.036	0.445	4.407*

* $p<0.05$, ** $p<0.01$, *** $p<0.001$

Table S3 Multivariate analysis results in LF-lacking mice.

Dependent variable	BW		TG		vLDL		R2	F
	B	SE	B	SE	B	SE		
Blautia	-0.003	0.001	-0.305*	0.042	0.103*	0.111	0.536	3.85*
NK4A214_group	1.842E-5	0	-0.045*	0.019	0.02*	0.007	0.586	4.725*
GCA-900066575	0	0	-0.008	0.021	0.024*	0.008	0.534	3.821*
Mucispirillum	-7.33E-05	0	0.022	0.01	0.007	0.004	0.577	4.545*
Peptococcus	6.955E-5	0	0.016**	0.005	0.005*	0.002	0.701	7.826**
Acetatifactor	-2.66E-05	0	0.001	0.002	0.002**	0.001	0.585	4.705*
A2	-1.48E-05	0	0.009***	0.002	0.002*	0.001	0.845	18.11****

* $p<0.05$, ** $p<0.01$, *** $p<0.001$

Table S4. Summary of changes in bacteria genera with the same or opposite trend in the three models.

genus	Age		HFD		DSS	
	wt-wt	ko-wt	wt-wt	ko-wt	wt-wt	ko-wt
Aerococcus	- -	↑ -	-	-	↓ -	↓ -
Alistipes	- -	- -	↑	-	↑ ↑	↑ ↑
Anaerotruncus	↓ ↓	↓ ↓	↑	↑	- ↓	↓ ↓
Bacteroides	↓ ↓	↓ ↓	-	-	↑ -	↑ ↑
Bilophila	↓ ↓	↓ ↓	↑	-	- -	- -
Blautia	↓ ↓	- -	↑	-	- -	- -
Candidatus_Arthromitus	↑ ↑	↑ ↑	-	-	↓ ↓	↓ ↓
Candidatus_Saccharimonas	↑ ↑	↑ ↑	-	-	↓ -	- ↓
Clostridium_sensu_stricto_1	- ↑	- ↑	↓	↓	↑ -	↑ -
Colidextribacter	- -	↓ ↓	↑	-	↑ -	↑ ↑
Corynebacterium	- -	↑ -	-	-	- -	↓ -
Desulfovibrio	- ↑	↑ ↑	-	-	- -	↓ ↓
Dubosiella	- -	↑ ↑	↓	↓	- ↑	- -
Enterococcus	↑ ↑	↑ ↑	-	-	- -	- ↑
Enterorhabdus	↑ ↑	↑ ↑	-	-	↓ ↓	↓ ↓
Eubacterium_brachy_group	- -	↑ ↑	-	-	↓ ↓	↓ ↓
Eubacterium_xylanophilum_group	↑ ↑	- -	↓	↓	↓ ↓	↓ ↓
GCA-900066575	↓ ↓	↓ ↓	-	↑	- ↓	↓ -
Jeotgalicoccus	↑ -	↑ -	-	-	↓ ↓	↓ -
Kurthia	↑ -	↑ -	-	-	↓ -	↓ -
Lachnoclostridium	- -	- ↑	-	↓	↓ ↓	↓ ↓
Lachnospiraceae_UCG-06	- ↑	↑ -	-	↓	- ↓	↓ -
Lactobacillus	- ↑	↑ ↑	-	-	↓ ↓	↓ -
Lysinibacillus	- -	↑ -	-	-	- -	↓ -
Microbacterium	- -	↑ -	-	-	- -	↓ -
Monoglobus	↑ ↑	- -	↓	-	↓ -	- -
Mucispirillum	↓ ↓	- -	↑	-	↑ ↑	↑ ↑
Muribaculum	↓ ↓	↓ ↓	↓	-	↓ -	↓ ↓
NK4A214_group	- -	- -	↑	↑	↓ -	↓ ↓
Odoribacter	- -	↓ ↓	↑	-	↑ ↑	↑ ↑
Oscillibacter	↓ ↓	↓ ↓	↑	-	↑ ↑	↑ ↑
Parabacteroides	↓ ↓	- -	-	-	↑ ↑	↑ ↑
Rikenella	↓ -	↓ -	↑	-	- -	↑ ↑
Romboutsia	↓ -	- -	-	-	↑ ↑	↑ -
Roseburia	↓ -	↓ -	↑	↑	↓ ↓	↓ ↓
Staphylococcus	↑ ↑	↑ -	-	↓	↑ ↓	↓ -
Turicibacter	↓ ↓	- -	↓	↓	↑ ↑	↑ ↑

"-", no change; " ↑ ", up regulate; " ↓ ", down regulate. Red arrow represents female mice, blue represent male mice.

Table S5 Genus with consistent impact trends on three models in the absence of LF during lactation.

	HFD	DSS	CUMS
Marvinbryantia	↑	↑	-
Atopostipes	↑	-	↑
Alistipes	↓	-	↓
Parasutterella	↓	-	↓
Rikenella	↓	-	↓

↑ : increased in ko-wt mice compared with wt-wt mice. ↓ : decreased in ko-wt mice compared with wt-wt mice.
